# Two responses to MeJA induction of R2R3-MYB transcription factors regulate flavonoid accumulation in *Glycyrrhiza uralensis* Fisch

**DOI:** 10.1371/journal.pone.0236565

**Published:** 2020-07-30

**Authors:** Yali Li, Xiuli Chen, Jiaqi Wang, Guangping Zou, Lu Wang, Xueshuang Li

**Affiliations:** 1 School of Life Science and Technology, Inner Mongolia University of Science and Technology, Baotou, China; 2 Baotou Teachers’ College, Biological Science and Technology Institute, Baotou, Inner Mongolia, China; Guangzhou University, CHINA

## Abstract

Flavonoids are key components of licorice plant that directly affect its medicinal quality. Importantly, the MYB family of transcription factors serves to regulate the synthesis of flavonoids in plants. The MYB transcription factors represent one of the largest families of transcription factors in plants and play important roles in the process of plant growth and development. MYB gene expression is induced by a number of plant hormones, including the lipid-based hormone jasmonate (JA). Methyl jasmonate (MeJA) is an endogenous plant growth regulator that can induce the JA signaling pathway, which functions to regulate the synthesis of secondary metabolites, including flavonoids. In this study, MeJA was added to licorice cell suspensions, and RNA-seq analysis was performed to identify the differentially expressed genes. As a result, the MYB transcription factors *GlMYB4 and GlMYB88* were demonstrated to respond significantly to MeJA induction. Subsequently, the *GlMYB4* and *GlMYB88* protein were shown to localize to the cell nucleus, and it was verified that *GlMYB4* and *GlMYB88* could positively regulate the synthesis of flavonoids in licorice cells. Overall, this research helps illustrate the molecular regulation of licorice flavonoid biosynthesis induced by MeJA.

## Introduction

Flavonoids are a class of naturally occurring compounds that have a 2-phenyl-chromone structure. A large number of studies have shown that flavonoids are non-toxic and harmless; in addition, they possess many biological activities and pharmacological effects, including antioxidant, anticancer, anti-inflammatory and so on [1–3]. Licorice (Glycyrrhiza uralensis Fisch) is mainly distributed in western China, including the Inner Mongolia, Ningxia, Xinjiang, and Gansu regions. It is one of the most widely used medicinal herbs, and both its roots and rhizomes are used therapeutically [4, 5]. The primary medicinal components of licorice are flavonoids and triterpenoids [6–9]. At present, licorice is widely used in both the pharmaceutical industry and in the production of food additives. In the latest research, the team of Liang Jiangong synthesized a kind of highly biocompatible CDs (Gly-CDs) from active ingredient (glycyrrhizic acid) of Chinese herbal medicine by a hydrothermal method, and found that the Gly-CDs possess extraordinary antiviral activity with multisite inhibition mechanisms for the porcine reproductive and respiratory syndrome virus (PRRSV) [10].

The biosynthesis of flavonoids in plants begins with the phenylpropane metabolic pathway [11, 12]. Among them, cinnamic acid-4-hydroxylase (C4H) and chalcone synthase (CHS) are two enzymes in the flavonoid synthesis pathway. C4H belongs to P450 monooxygenase, which acts on the second step of the whole metabolic pathway and is a key enzyme in the phenylpropane metabolic pathway. The protein activity of C4H has a direct effect on the synthesis of flavonoids, lignin, etc., and is an important adjustment point in the metabolism of phenylpropanoids. CHS is a key enzyme that leads the phenylpropane metabolic pathway to the synthesis of flavonoids. MYB transcription factors (TFs) can affect the expression of *CHS* gene.

The MYB TFs comprise one of the largest families of TFs in plants and play important roles in the processes of plant growth and development. Additionally, these are the main TFs involved in the regulation of flavonoid synthesis [13–16]. In the metabolic pathway of flavonoids, including cell wall component synthesis, biosynthesis of glucosinolates, other primary and secondary metabolic reactions, they also play an important role in metabolic regulation [17–20]. Studies have shown that *MYB* genes can be induced by environmental factors, such as ABA (abscisic acid), SA (salicylic acid), jasmonate (JA), and others [21–24].

Methyl jasmonate (MeJA), which is a JA analog, is one of the main signal molecules in the phenylpropane metabolic pathway [25]. Studies have shown that JA signaling has a regulatory effect on the synthesis of secondary metabolites, such as terpenoids, phenylpropanoids, and alkaloids, which present with a wide range of biological functions [26]. Some secondary metabolites have been shown to accumulate in plant cells following MeJA treatment, including paclitaxel in Taxus cells [27, 28], terpenoid in *Centella asiatica* cells [29], and ginseng saponins in ginseng cells [30]. However, the biological mechanism underlying the induction of licorice flavonoids by MeJA and the associated changes in the transcriptome are relatively unknown.

In the present study, changes in the transcriptional profile in licorice cells were assessed following MeJA treatment via the RNA-Seq method. In total, differentially expressed MYB TFs were identified based on this analysis. The *GlMYB4* and *GlMYB88* gene were subsequently cloned. As these genes presented significantly altered MeJA-induced expression, and the effect of *GlMYB88* was obvious. Based on bioinformatic analyses, subcellular localization, overexpression, and flavonoid accumulation, it was verified that these genes functioned as a flavonoid synthesis-related gene in licorice. These results help to illustrate the molecular regulatory mechanisms underlying the biosynthesis of licorice flavonoids induced by MeJA. Moreover, this study serves as a valuable resource for further analysis of the regulatory mechanisms involved in flavonoid biosynthesis in plants.

## Materials and methods

### Plant materials and reagents

The experiment was carried out in the plant cell culture room of the Inner Mongolia University of Science and Technology (China). The tested licorice variety was *Glycyrrhiza uralensis* Fisch, and the seeds were purchased from Erdos City, Inner Mongolia, China. A licorice cell suspension culture system was established, and the entire operation was carried out at 25±1°C, with the pH maintained at 5.8 [5]. *Nicotiana benthamiana* was grown in a glasshouse and used for subcellular localization analysis.

Some kits, antibiotics, pUCm-T vectors, and primers used in the experiment were purchased from Sangon Biotech (Shanghai, China). Restriction enzymes were purchased from Takara Biomedical Technology (Beijing, China). T4 DNA Ligase, ampicillin, 6-benzyladenine (6-BA), naphthaleneacetic acid (NAA), 2,4-dichlorophenoxyacetic acid (2,4-D), and MeJA were purchased from Sigma-Aldrich (USA). *Escherichia coli* DH5α, *Escherichia coli* Top10, *Agrobacterium tumefaciens* GV3101, and pJG054 plasmid vectors were all available and stored in the laboratory.

### Establishment of cell suspension culture system and MeJA treatment

The calli were induced from the young hypocotyl of licorice aseptic seedlings, from which the licorice cells were obtained. After culturing for 20 generations on a solid medium, the cells were inoculated into 100 mL of MS liquid medium supplemented with 2,4-dichlorophenoxyacetic acid (2,4-D, 1.0 mg•L^-1^), naphthaleneacetic acid (NAA, 0.5 mg•L^-1^), and 6-benzyladenine (6-BA, 0.5 mg•L^-1^) and then cultured at 120 rpm for 3 weeks (S1 Fig) [5]. A well-grown suspension cell line was selected, cleaned with liquid medium, and then transferred to a fresh suspension medium containing 100 mL MeJA (LABEST, China) at a final concentration of 100 uM. Alternatively, an equal volume of anhydrous ethanol was added to the liquid medium as a control. Then, the cells were cultured at 120 rpm for 48 h. The treatment method is shown in S1 Table.

### RNA extraction, cDNA acquisition, and qPCR

RNA from filtered cells was extracted using RNAiso Blood extraction reagent (Takara, China). cDNA acquisition was achieved using a PrimeScript RT reagent kit with gDNA Eraser (Takara, China). The primers used for the *CHS*, *C4H*, and *Actin* genes are presented in S2 Table. qPCR was performed using TB Green Premix Ex Taq (Takara, China), and run on an ABI7500 Real-Time PCR machine (ABI, USA) following the manual’s recommendations. Then the resulting data were analyzed using the 2^(-ΔΔCt) method [31].

### Sequencing

After analyzing the cells in each group from the above experiments, we selected an experimental group in which the relative expression levels of the two enzyme genes changed significantly. The suspension cultured cells were selected which treatment by MeJA and filtered, the constant weight cells were sequenced in Beijing novogene company (China).

In this research, it is mainly analyzed about gene expression level, genetic function commentary, RNA-seq quality evaluation and differential expression. Among them, the genetic function commentary includes RefSeq non-redundant proteins (NR), Gene Ontology (GO), the Kyoto Encyclopedia of Genes and Genomes (KEGG).

### Gene cloning and vector construction

RNA-seq was used to screen MYB TFs significantly responding to MeJA induction, and the predicted sequences were obtained, which were named *GlMYB88* and *GlMYB4*. The cloning of *GlMYB4* and *GlMYB88* was performed using a TransStart FastPfu DNA Polymerase kit (Trans, China); the primers used are presented in S2 Table. The amplification period was: 95°C 20 s, 58°C 20 s, 72°C 1 min for 40 cycles. The PCR product was ligated with the pUCm-T vector at 16°C overnight, generating the T-*GlMYB88* and T-*GlMYB4* vector, and then transferred into *E*. *coli* Top10 sensitive cells. The positive clones were screened and sent to Sangon Biotech (Shanghai, China) for sequencing. Retrieve the results through the Pfam online database (http://pfam.sanger.ac.uk/). The protein sequences were put into the NCBI database for comparison, and MEGA7.0 software was used to construct the phylogenetic tree and analyze the genetic relationship.

For the construction of the expression vector pJG054-*GlMYB88* and pJG054-*GlMYB4*, the T-*GlMYB88* and T-*GlMYB4* plasmid were used as the template for PCR using the J88-F/R and J4-F/R primers, which are presented in S2 Table. The amplification sequence was ligated with *Apa*I-digested pJG054 vector to generate a GlMYBs-YFP fusion construct under the control of cauliflower mosaic virus 35S (CaMV 35S) promoter, then transformed into *E*. *coli* Top10 sensitive cells. The positive clones were screened.

### Subcellular localization analysis

The recombinant plasmid pJG054-*GlMYB88* and pJG054-*GlMYB4* were extracted and transferred into *Agrobacterium* GV3101 competent cells, which were then cultured at 28°C for 48 h. The positive colonies were screened and injected into tobacco leaves (6 weeks old) via the injection osmotic method. Fluorescence was then observed under a laser confocal microscope (Nikon, Japan) after 60–72 h of culture.

### Expression analysis of *GlMYB4* and *GlMYB88* in the different structures and growth periods of licorice

RNA was extracted from root, stem, leaf, and cotyledon samples of aseptic licorice seedling at different growth periods (2, 3, 4 and 5 weeks) to obtain cDNA (methods detailed above). The primers M88D1-F/R and M4D1-F/R (S2 Table) were used, and qPCR was performed. The Actin reference gene served as a control to analyze the relative expression of *GlMYB4* and *GlMYB88* in these different structures and at the different growth periods.

### Overexpression of *GlMYB4 and GlMYB88* in licorice cells

Since the selected vector pJG054 contains kanamycin resistance gene, in order to ensure that the transformed cells can grow normally, it is necessary to screen for the kanamycin resistance concentration of licorice cells. Licorice cells in good growth condition were inoculated on cell which cultured on solid media culture medium with different kanamycin concentration, cultured in light incubator for 30 days, and their growth status was observed.

*Agrobacterium* harboring the pJG054-*GlMYB88* and pJG054-*GlMYB4* plasmid were co-cultured with suspended licorice cells for 2 days. The genomic DNA of the cells was extracted by the CTAB method to detect vector marker gene in transformed cell [32]. Total RNA was then extracted from transformed cell for qPCR validation (methods detailed above). The remaining cells were subcultured. After 40 days, the licorice cells were dried and ground to a powder, and 20 times the volume of 80% methanol was added to soak for 24 hours. The total flavonoids in the cells were extracted via an ultrasonic method, and the flavonoid content was detected using UV-Vis spectrophotometry (510 nm) [33]. The content of isoliquiritigenin was detected by High Performance Liquid Chromatography (HPLC).

## Results

### Expression of the *CHS* and *C4H* genes in response to MeJA induction

**Fig 1:**
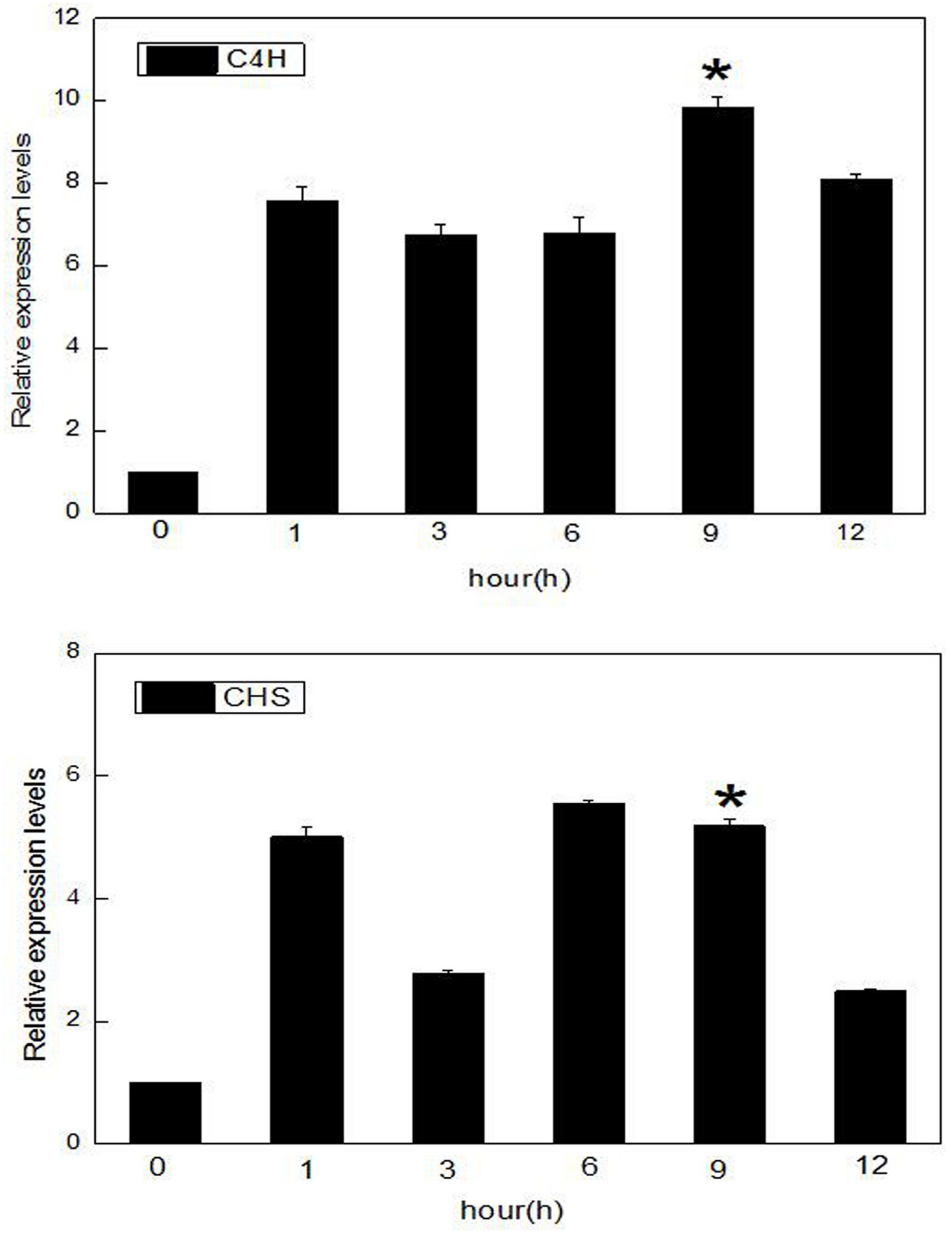
Expression analysis of the *CHS* and *C4H* genes in licorice cells. Data presented here are the mean of three replicates with error bars indicating ± SD. Asterisks indicate significant differences with values for the untreated cell: *P < 0.05. The expression levels of *CHS* and *C4H* in cells treated with MeJA at different times as based on qPCR analysis; *C4H*: cinnamic acid-4-hydroxylase; *CHS*: chalcone synthase.

More than 20 generations of cells were selected for the establishment of the suspended cell lines, and a suspension of free cells was formed following 2–3 subcultures (S1 Fig). Total RNA was extracted from the cells following the different treatment conditions. qPCR experiments were conducted using a quantitative method, and these experiments were each repeated three times. The results showed that the relative expression levels of the *C4H* and *CHS* genes, both known flavonoids biosynthesis genes, in the MeJA treatment group were higher than those in the control group within 1–12 h, and their relative expression levels were the highest after 9 h (Fig 1). This effect was significant compared with the other treatment times. Overall, the relative expression of the *C4H* gene was generally greater than that of the *CHS* gene, and these results indicated that MeJA could regulate the expression of both of these genes.

### Functional genetic analysis

The expression of *C4H* and *CHS* was the highest after 9 h of MeJA induction. As such, cells at this time point were selected for library building and Illumina sequencing. From the statistical table of the splicing of transcripts, the transcripts have 151,529 and unigenes have 116,07 (S3 Table). The transcriptome datasets derived from these licorice cells were submitted to the Gene Expression Omnibus (GEO) (number GSE128503).

**Fig 2:**
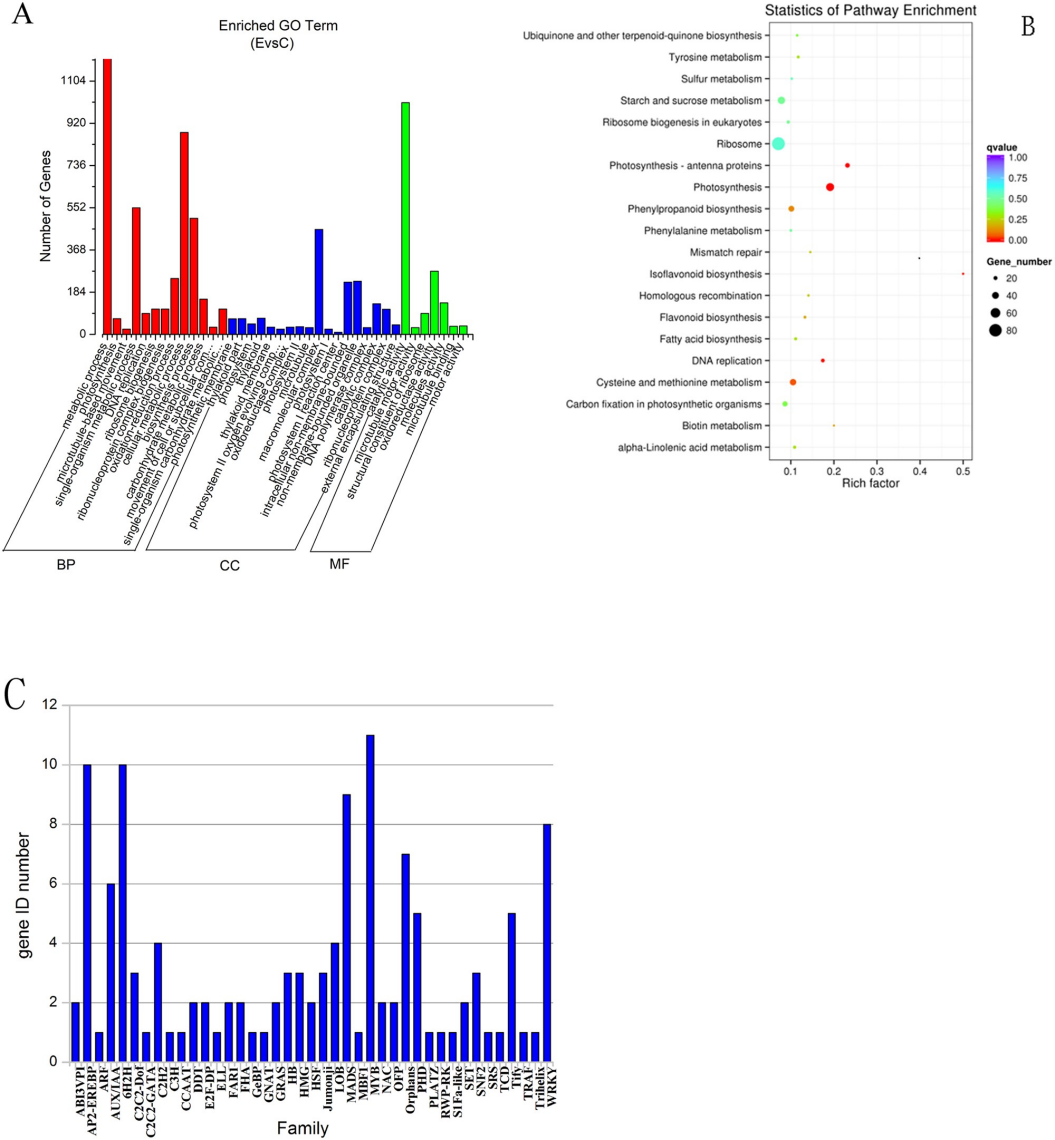
Sequence data analysis. (A) Enriched GO Terms. Find out which of the different genes are higher in the GO group. (B) KEGG pathway enrichment diagram depicting the differentially expressed genes (DEGs) among the metabolic pathways. (C) Classification of the genes with significant differential expression. Identity of the genes with the most apparent changes in their expression levels.

Functional annotation of the licorice transcriptome sequences was performed using the NR database available at NCBI. The NR database is a protein database that is generally used to annotate protein function and species. By comparing the experimentally obtained gene sequences with the NR database, the similarity between the gene sequence of the experimental species and the gene sequence of related species, as well as the associated functional information, can be obtained. Based on the results of the NR library comparison, the sample sequences appeared to be most similar to those of legume plants (S2 Fig). GO analysis evaluates the target genes based on three major categories: biological process, molecular function, and cell composition/localization. A total of 35,556 unigenes were successfully annotated via GO, covering up to 30.41% of the total unigenes. Using RSEM software to analyze the gene expression levels, a total of 2,650 differentially expressed genes (DEGs) were identified, with 1,464 genes shown to be up-regulated and 1,186 genes shown to be down-regulated (S3 Fig). By mapping all the DEGs to each term of the GO database, the most highly enriched GO functional items were identified among the DEGs in comparison with the genomic background. This analysis indicated that many of the DEGs were significantly related to known biological functions (Fig 2A), with most of the DEGs dominant in five terms, including cellular process, metabolic process, single-organism process, binding, and catalytic activity.

The KEGG database is used to systematically analyze the metabolic pathways associated with gene products and compounds in the cells and helps establish the functions of the target genes by integrating genome, chemical, molecular, and biochemical systems data. To search for metabolic and signal transduction-related genes, all of the DEGs present in the KEGG database were mapped. The KEGG enrichment analysis results were converted into scatter diagrams (Fig 2B), and the rich factor in this figure represents the ratio of the number of genes under the pathway entry among the DEGs relative to the total number of genes for the pathway entry among all the annotated genes. The closer the q-value is to 0, the more significant the enrichment is. Overall, the results indicated that the gene change for the isoflavonoid biosynthesis pathway was most apparent and that the enrichment degree was large.

TFs regulate the spatiotemporal expression of plant defense genes in response to abiotic and biotic stresses. This analysis showed that the DEGs that responded to MeJA elicitation were largely represented by the TF families regulating secondary metabolism and stress responses in plants, including the AP2-EREBP, bHLH, MADS, MYB, and WRKY families (Fig 2C). The most of up-regulated TFs were MYB TFs, which are known to play key roles in MeJA-mediated flavonoids biosynthesis and the MeJA response network in licorice cells.

### Cloning and bioinformatic analyses of *GlMYB4* and *GlMYB88*

**Fig 3:**
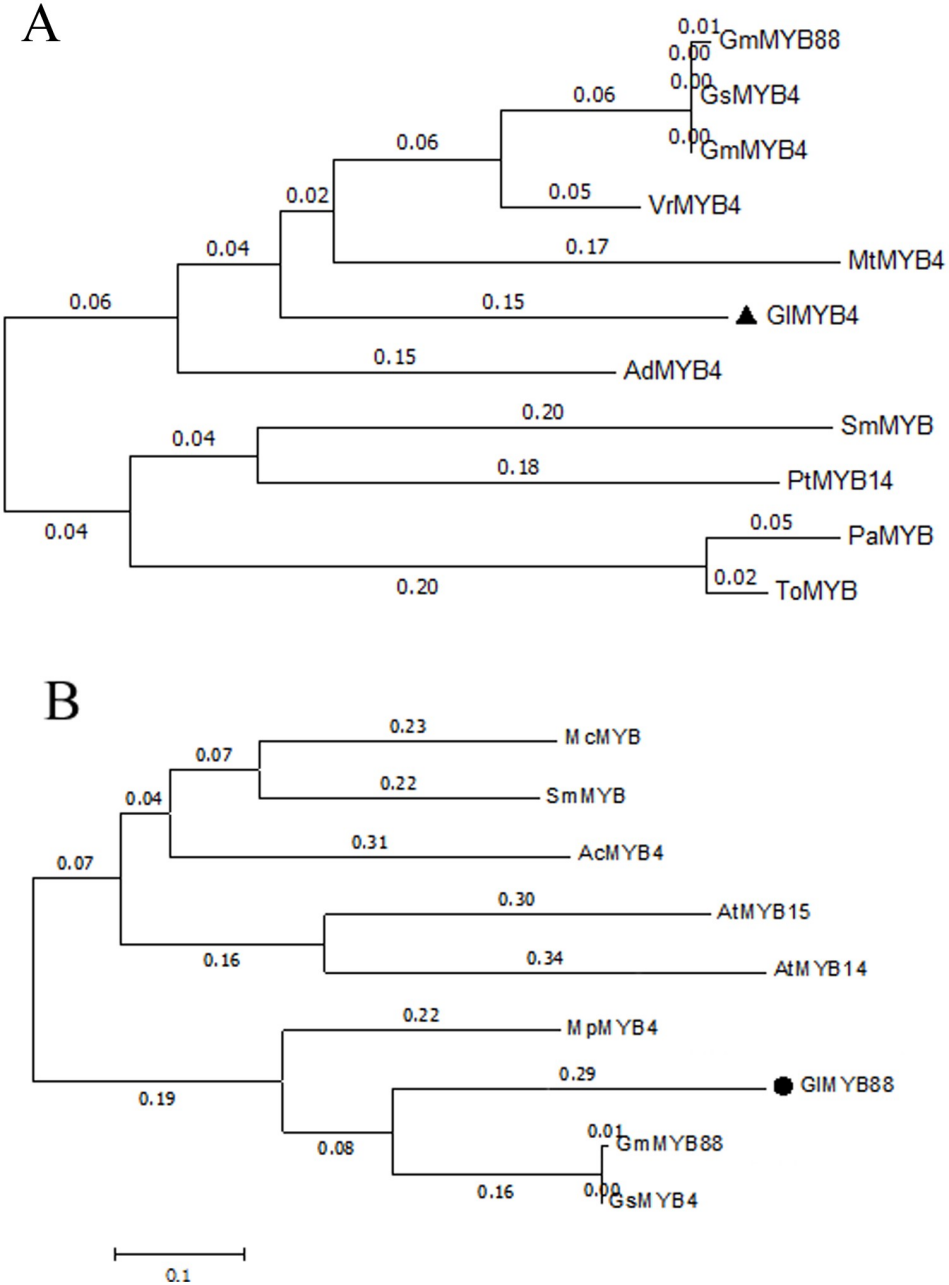
*GlMYBs* phylogenetic tree. (A) *GlMYB4* phylogenetic tree. (B) *GlMYB88* phylogenetic tree.

The *GlMYB4* gene was cloned (S4A Fig) and sequenced, and the sequencing results were compiled using DNAMAN software. The cDNA sequence contained a 753 bp ORF encoding a total of 251 amino acids. The *GlMYB4* gene sequence was submitted to GenBank for sequence alignment, and the related TF protein sequences from other plants were downloaded. The phylogenetic tree was constructed using MEGA7.0 software. A shown in Fig 3A, several MYB genes were identified that are closely related to the typical R2R3-MYB TF. The genetic relationship between *GlMYB4* and *VrMYB4*, *GsMYB4*, *GmMYB4*, and *GmMYB88* are very close, and they all harbor the typical R2R3 structure.

The *GlMYB88* gene was cloned (S4B Fig) and sequenced. The cDNA sequence contained a 1077 bp ORF encoding a total of 359 amino acids. The phylogenetic tree was constructed using MEGA7.0 software. A shown in Fig 3B, several MYB genes were identified that are closely related to the typical R2R3-MYB TF. The genetic relationship between *GlMYB88* and *GmMYB88*, *GsMYB88*, *MpMYB4* are very close, and they all harbor the typical R2R3 structure.

### Subcellular localization of *GlMYB4* and *GlMYB88*

**Fig 4:**
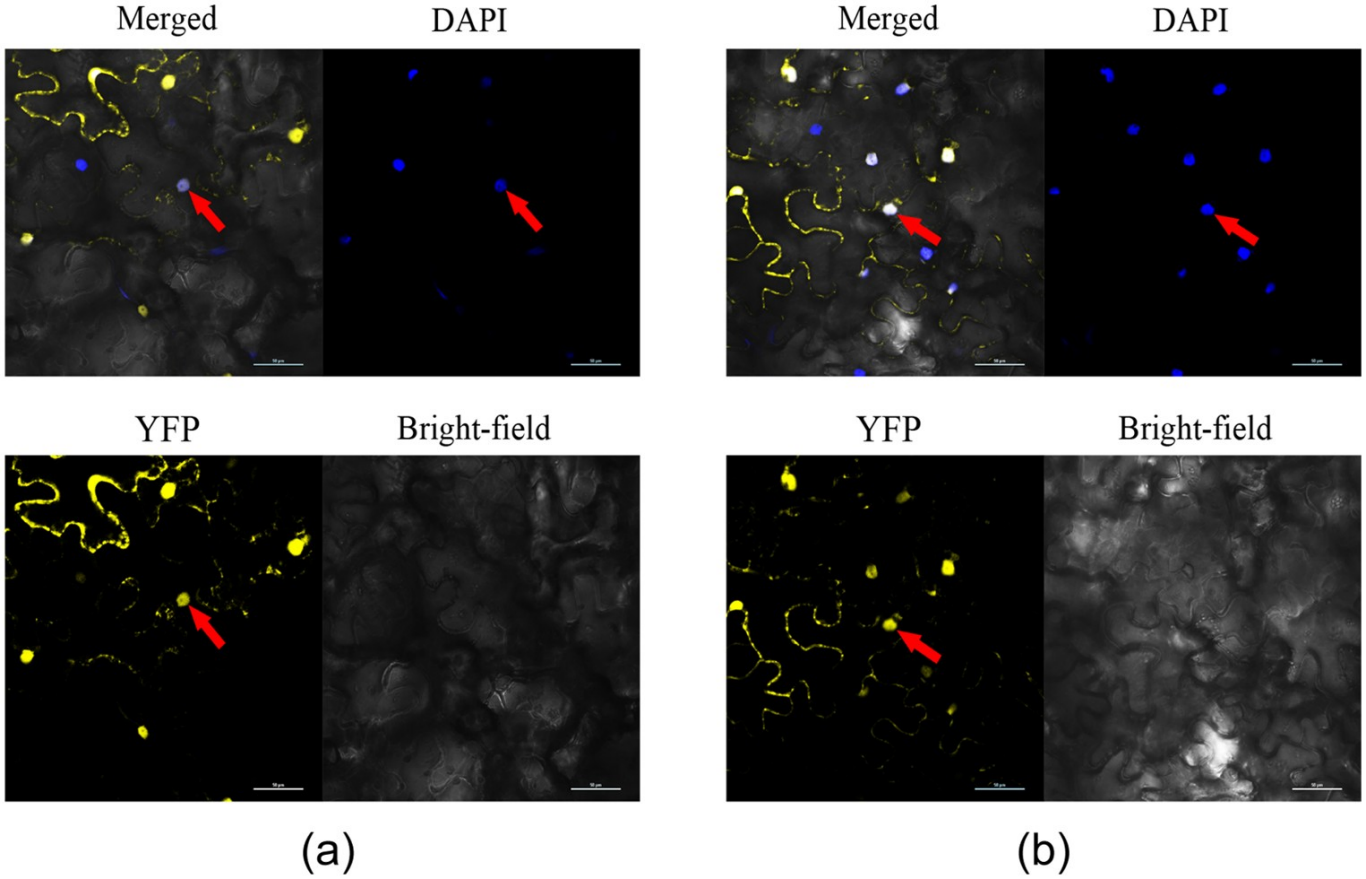
GlMYBs subcellular localization analysis. DAPI is used as the nuclear dye, and YFP is the fluorescent protein carried by the target gene. Bright-field image of the cellular morphology under unexcited light. Merged represents the overlapping of all three images. The red arrow indicates the position of the nucleus and GlMYBs protein co-localization. (A) GlMYB4 subcellular localization analysis. (B) GlMYB88 subcellular localization analysis.

The linearized pJG054 vector was ligated to the *GlMYB4* PCR product to construct the recombinant expression plasmid pJG054-*GlMYB4* (S5 Fig), which was transferred into *Agrobacterium* GV3101 competent cells. Tobacco leaves were then injected with the transformed *Agrobacterium* and observed under laser confocal fluorescence microscope after 60 h. As shown in Fig 6, the yellow fluorescence carried by the recombinant vector p054-*GlMYB4* overlaps with the DAPI blue fluorescence showing the nuclear position, then it appears white. It was found that the expressed GlMYB4 protein was localized to the nucleus (Fig 4A). Similarly, *GlMYB88* protein is located in the nucleus (Fig 4B).

### Expression analysis of *GlMYB4* and *GlMYB88* in different structures and growth periods of licorice

**Fig 5:**
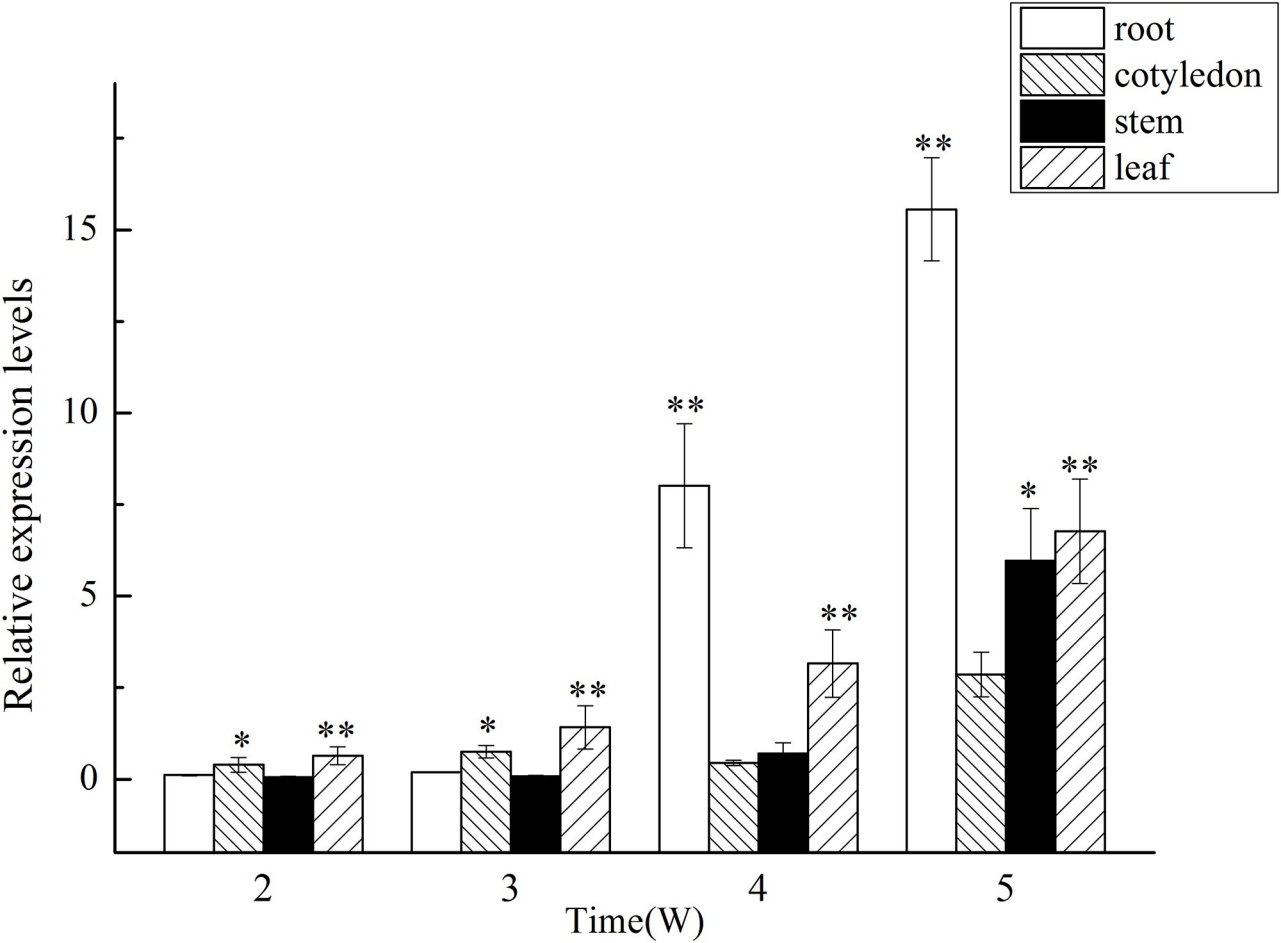
Expression analysis of *GlMYB4* in different structures and growth periods of licorice. Data presented here are the mean of three replicates with error bars indicating ± SD. Asterisks indicate significant differences with values for aseptic licorice seedling at different structures and growth periods: *P < 0.05; **P < 0.01. (A) Expression analysis of *GlMYB4* in different growth periods of licorice. (B) Expression analysis of *GlMYB4* in different structures of licorice.

**Fig 6:**
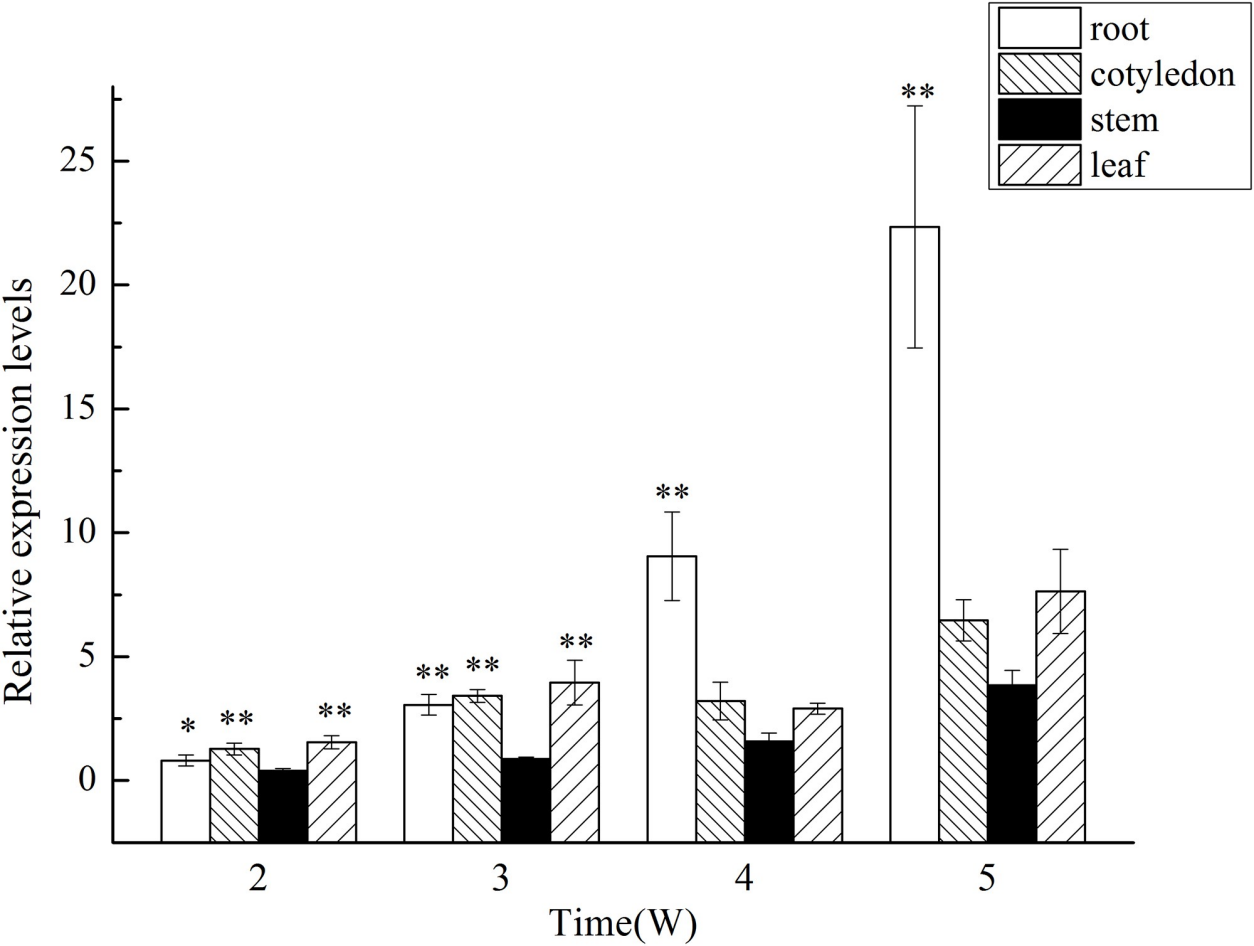
Expression analysis of *GlMYB88* in different structures and growth periods of licorice. Data presented here are the mean of three replicates with error bars indicating ± SD. Asterisks indicate significant differences with values for aseptic licorice seedling at different structures and growth periods: *P < 0.05; **P < 0.01. (A) Expression analysis of *GlMYB88* in different growth periods of licorice. (B) Expression analysis of *GlMYB88* in different structures of licorice.

To determine the expression of *GlMYB4* and *GlMYB88* in the different structures and growth periods of licorice plants, tube seedlings at 2, 3, 4, and 5 weeks of age were assessed via qPCR (Figs 5 and 6). The results showed that the expression of *GlMYB4* and *GlMYB88* changed substantially over time. As shown in Fig 5, at 2 weeks, the seedlings had just grown up, and there was no significant difference in the expression levels of *GlMYB4* in roots, cotyledons, stems, and leaves. From 3 weeks, *GlMYB4* gene expression level began to differ in different organs, but the difference was not significant. However, the expression level in the roots gradually increased and exceeded the expression level in the leaves at 4 weeks. At this time, the cotyledons began to turn yellow from the edge, and the leaves grew more vigorously, and the roots grew continuously in the medium. At 5 weeks, the roots demonstrated more growth activity, while the cotyledons began to wither. Additionally, at this time point, the leaves also began to undergo yellowing and *GlMYB4* expression was highest in the roots. The expression level of *GlMYB88* is basically similar to that of *GlMYB4* (Fig 6). In general, the relative expression levels of *GlMYB4* and *GlMYB88* increase with the growth period of the plant, and the expression level of *GlMYB88* was higher than that of *GlMYB4*.

### Overexpression of *GlMYB4* and *GlMYB88*

As the recombinant vector contained kanamycin resistance gene, so kanamycin-resistant concentration were screened before transformation experiments. In the experiment, licorice cells was inoculated with different kanamycin concentration medium (10–150 mg•L^-1^) for 30 days. When the kanamycin concentration was 50 mg•L^-1^, the cells were able to grow normally within 30 days (S6 Fig). Combined with the growth conditions of the transformed *Agrobacterium*, 50 mg•L^-1^ was selected as the concentration used in subsequent experiments. When the exogenous gene p054-*GlMYBs* was inserted and integrated into the genome of licorice cells, the gene sequence on the recombinant vector could be amplified by PCR. Genomic DNA was extracted from the transgenic cells and using the untransgenic cell as the control, and specific primers were designed for the amplification of a gene segment with the YFP gene sequence on the plasmid. The results showed that the recombinant plasmid was successfully integrated into the genome of licorice cell (S7 Fig).

**Fig 7:**
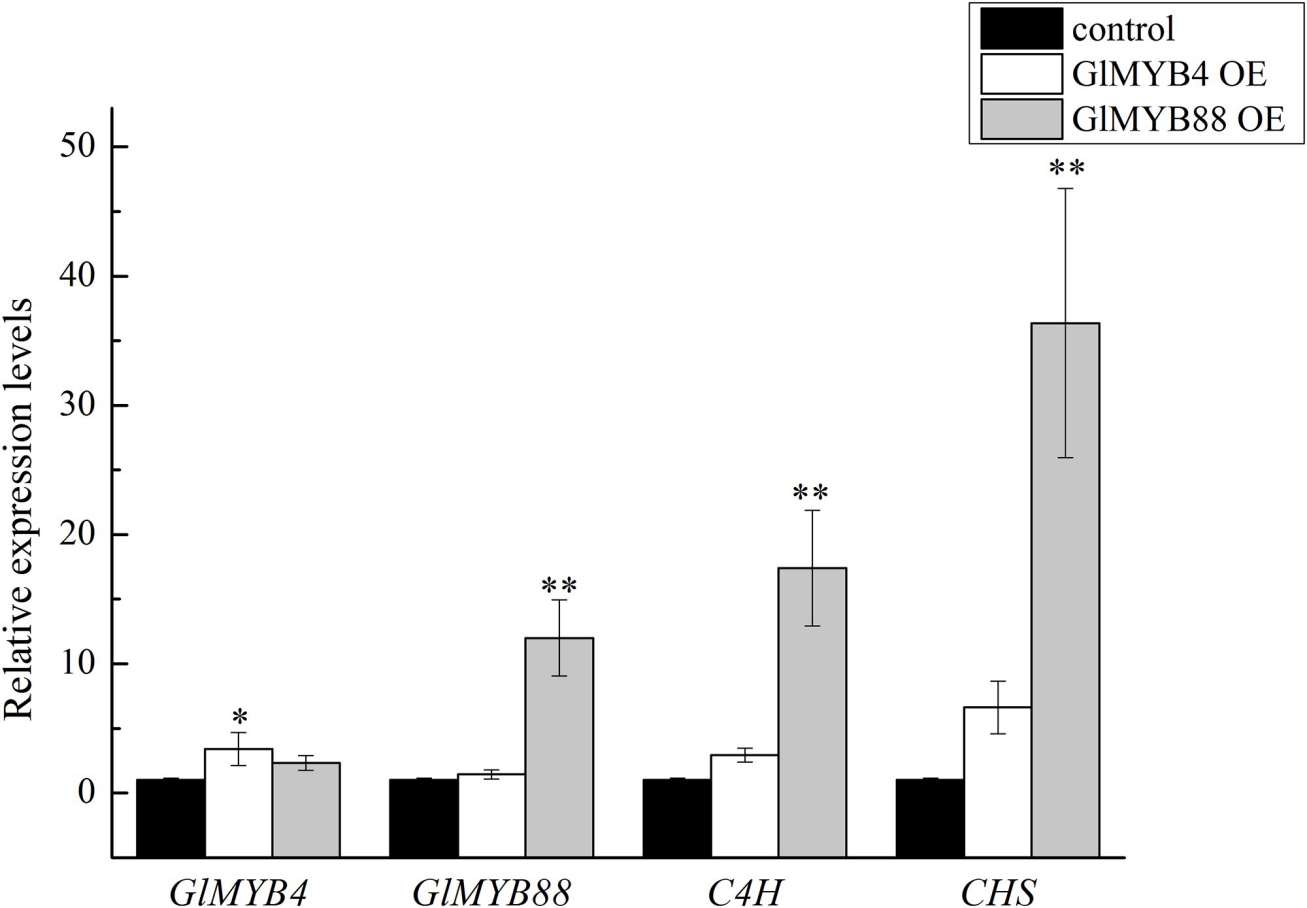
The expression level of the *GlMYB4*, *GlMYB88*, *C4S*, and *CHS* genes in the control and overexpression (OE) cells. Data presented here are the mean of three replicates with error bars indicating ± SD. Asterisks indicate significant differences with values for the empty vector control: *P < 0.05; **P < 0.01. Control: untransformed cell; GlMYB4 OE: overexpression *GlMYB4* group; GlMYB88 OE: overexpression *GlMYB88* group; *C4H*: cinnamic acid-4-hydroxylase; *CHS*: chalcone synthase.

The expression level of the *GlMYB4* gene in the suspended licorice cells harboring the overexpression *GlMYB4* plasmid was significantly higher than that in the control group, as based on qPCR analysis, and the expression levels of the *CHS* and *C4H* genes were also shown to be significantly higher than the control group (Fig 7) in the. The results of *GlMYB88* were similar to that of *GlMYB4*, and the effect of *GlMYB88* was more significant.

**Fig 8:**
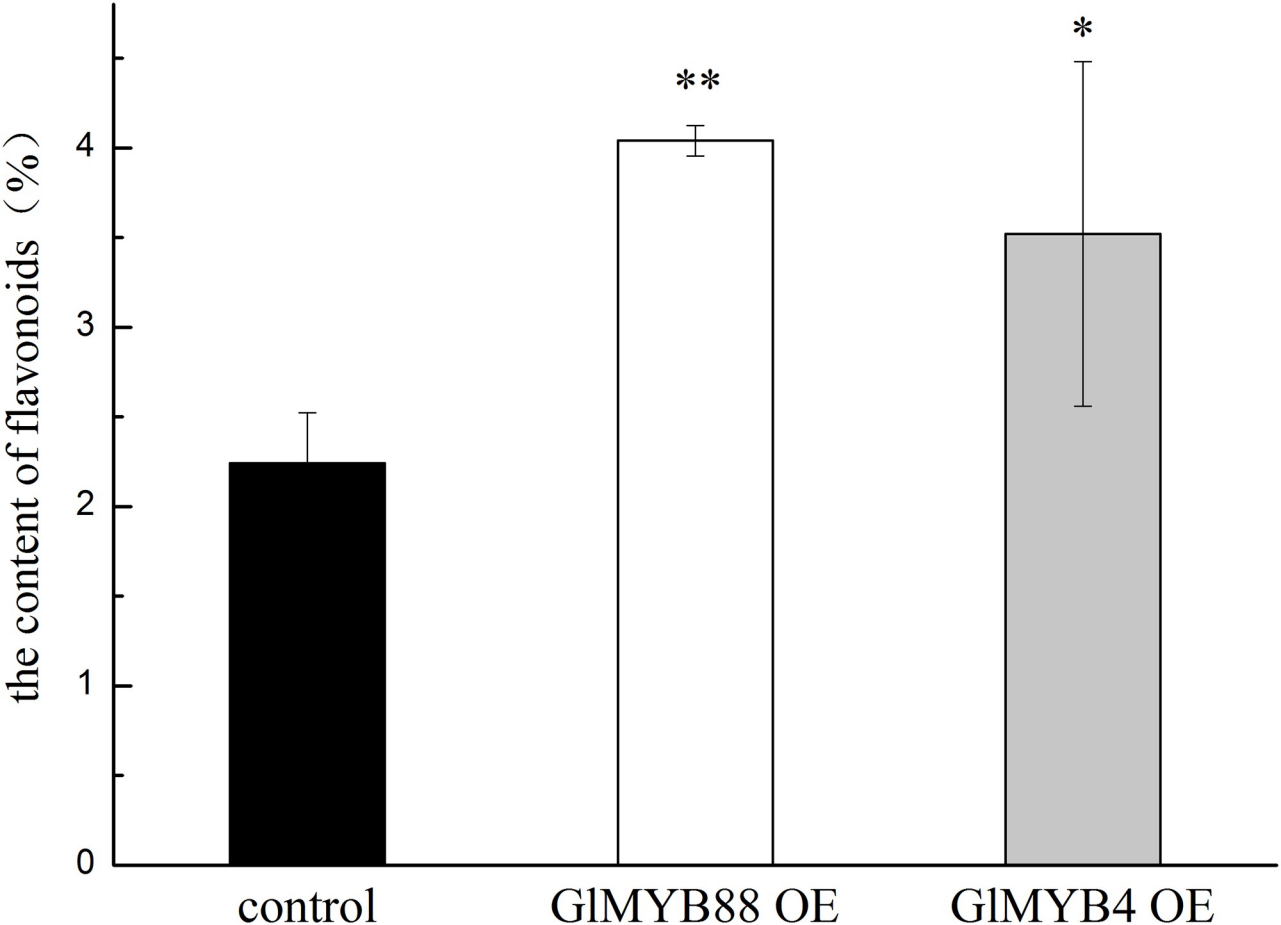
Determination of the total flavonoids in the control and experimental groups. Data presented here are the mean of three replicates with error bars indicating ± SD. Asterisks indicate significant differences with values for the empty vector control: *P < 0.05; **P < 0.01. Control: untransformed cell; GlMYB4 OE: overexpression *GlMYB4* group; GlMYB88 OE: overexpression *GlMYB88* group.

**Fig 9:**
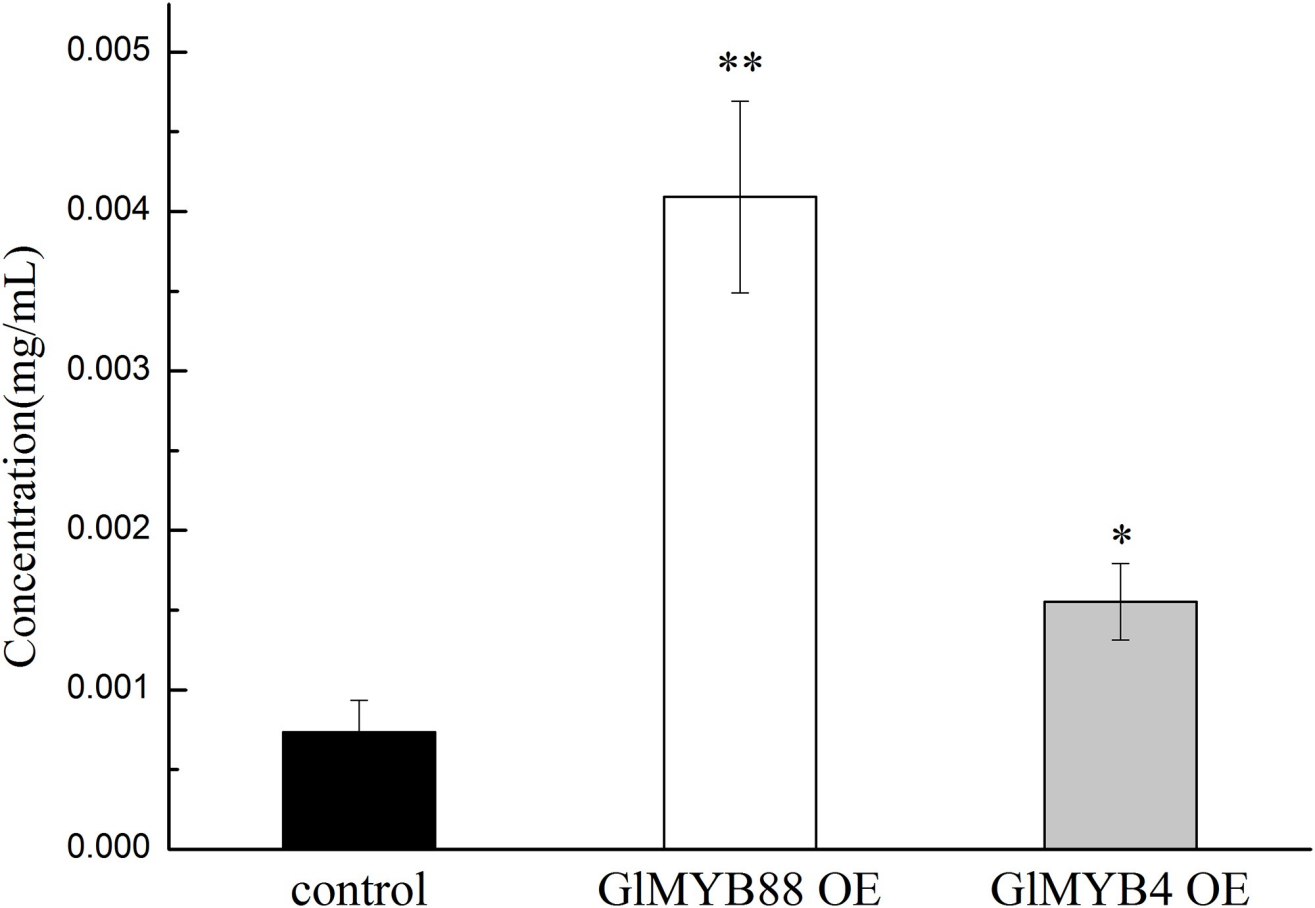
Determination of the isoliquiritigenin in the control and experimental groups. Data presented here are the mean of three replicates with error bars indicating ± SD. Asterisks indicate significant differences with values for the empty vector control: *P < 0.05; **P < 0.01. Control: untransformed cell; GlMYB4 OE: overexpression *GlMYB4* group; GlMYB88 OE: overexpression *GlMYB88* group.

In these cells, the total amount of flavonoids was measured via UV-Vis analysis after culturing over 40 days, and it was found that the content of flavonoids in the overexpression *GlMYB4* group was 1.57 times that of the control. The content of flavonoids in the overexpression *GlMYB88* group was 1.80 times that of the control (Fig 8 and S8 Fig). Isoliquiritigenin is a typical monomer of licorice flavonoids. The content of isoliquiritigenin in each group of cells was detected by HPLC (Fig 9 and S9 Fig). The results showed that the content of isoliquiritigenin increased in the transgenic cells. Among them, the content of isoliquiritigenin in the transgenic *GlMYB88* cell line was the highest, about 5.57 times that of the control group; the content of isoliquiritigenin in the transgenic *GlMYB4* cell line was about 2.11 times the control group.

## Discussion

When licorice cells are induced by MeJA, the overall yield of flavonoids can be increased [34]. In addition to licorice, there are many studies showing that the exogenous MeJA can induce the JA signal pathway in plants, including *Arabidopsis*, Taxus cells, and *Centella asiatica* [12, 27, 29, 30, 35–37]. When the exogenous hormone MeJA enters the plant, MeJA is converted to JA via the action of jasmonic acid carboxyl methyl transferase (JMT), thus increasing the JA content, which can complex with Ile to form JA-Ile. Active JA-Ile can induce SCF^COI1^-mediated degradation of Jasmonate ZIM-domain proteins (JAZ) and the JA-responsive TF is freed to play a regulatory role in downstream gene expression, thus initiating the plant's response to external environmental stressors [26, 37, 38]. At present, the TFs involved in the synthesis of plant secondary metabolites and the response to JA signaling primarily include ERF, bHLH, MYB, WRKY, HD-ZIP, DOF, NAC, and TFIIIA zinc fingers.

Studies have shown that the biosynthesis of flavonoids is regulated by MYB transcription factors [20, 39–43]. The MYB gene sequence was first identified by Graf in 1941 from AMV and E26, which caused avian acute myeloblastic leukemia viruses. In 1982, Klempnauer and other researchers identified a common transforming gene, called v-myb oncogene, from the avian myeloma virus. Clorless1 (C1) of maize was the first MYB transcription factor isolated and identified in plants [44–46]. Based on the number of MYB domain repeats (R), plant MYB proteins are divided into four sub-categories: 1R-MYB (containing only one R), R2R3-MYB, R1R2R3-MYB, and 4R-MYB (each containing four similar R1/R2 repeats). Among these, R2R3-MYB represents the largest class of MYB TFs in plants. MeJA can regulate the binding of MYB TFs to specific upstream DNA sequences and induce the efficient synthesis of plant secondary metabolites [47]. A study of 125 R2R3-MYB members demonstrated that about 32% of the members could be induced by JA, with about 4% down-regulated under these conditions [48]. MYB proteins with different functions were isolated and identified from plants such as arabidopsis thaliana and soybean and studied [17, 18]: *AtMYB24* played an important role in the development of *arabidopsis thaliana*;*GmMYBJ3* could increase the biosynthesis of isoflavones in soybean. These studies indicate that the MYB TFs are extensively involved in hormonal response processes, which play important roles in regulating the plant stress response. For legumes, studies on MYB TFs are more common in some common species such as soybean, and there are also studies on astragalus and pueraria, but there are few studies on MYB transcription factors in licorice. RNA-Seq is a rapid and efficient method for analyzing gene expression in tissues and cells and, as such, can make an important contribution to the understanding of the molecular mechanisms in plants [49–52]. Moreover, this method can detect low-abundant transcripts [53–55] and provides important information related to gene expression and transcriptional profiles [54–56]. In this study, transcriptome sequencing of licorice cells after MeJA treatment was performed, and a total of 11 MYB genes were identified that responded significantly to MeJA induction. Subsequently, *GlMYB4* and *GlMYB88* were cloned and analyzed.

Licorice has a variety of pharmacological activities, including antiviral, anticarcinogen and other effects [57–60], and this plant is widely used as one type of tonic class of medicinal materials. In practical application, the parts of the licorice plant that are typically used medicinally are mainly the roots and rhizomes [60]. A large number of studies have also shown that the main active ingredients in licorice accumulate in the roots more than other parts. TFs act on the upstream of specific target genes to regulate gene expression, and their expression level in the body should also vary with the level of target gene expression. In this study, the quantitative expression of *GlMYB4* and *GlMYB88* in the different structures and growth periods of licorice was analyzed. It was shown that, although the expression level of *GlMYB4* and *GlMYB88* were not high in the roots at the early stage of tube seedlings, their expression were gradually increased as the plants grew, indicating that expression level of *GlMYB4* and *GlMYB88* can respond to different accumulation of flavonoids.

Proteins are the main carriers of life activities, and genetic material needs to be translated into proteins to function. There are various organelles (or substructures) in the cell, which together constitute the most basic structure of the cell and enable the cell to function normally. Subcellular localization technology can provide important reference information for determining the function of some unknown proteins. A large number of studies have shown that most transcription factors are located in the nucleus, but a few transcription factors are found in cytoplasm, cell membrane and so on. We subcellularly localized the proteins encoded by *GlMYB4* and *GlMYB88*, the experimental results showed that *GlMYB4* and *GlMY88* proteins were shown to localize to the nucleus via laser scanning confocal microscopy.

Flavonoids are secondary metabolites of licorice and are important medicinal ingredients. Most flavonoids are derived from the phenylpropane metabolic pathway, in which phenylaline is converted to cinnamic acid by phenylalanine aminolase (PAL) catalysis, and cinnamic acid is catalyzed by C4H to produce 4-hydroxycinnamic acid. Subsequently, 4-hydroxycinnamic acid is transformed into coumaryl-coenzyme A by 4-coumaryl-CoA synthase (4CL). One molecule of 4-coumaryl-CoA and three molecules of malonyl-CoA can be synthesized into charketone under the action of CHS, and a variety of flavonoids can be derived after charketone is catalyzed by chalcone isomerase (CHI) [11, 12]. In this study, expression level of two key enzyme genes, *C4H* and *CHS*, were analyzed to verify that *GlMYB88* and *GlMYB4* can regulate the flavonoid synthesis in licorice. When *GlMYB4* and *GlMYB88* were overexpressed, the expression levels of the key enzyme genes *CHS* and *C4H* were also significantly increased, and the total flavonoid and isoliquiritigenin content were also increased. These observations indicated that *GlMYB*4 and *GlMYB88* likely functioned as a TF that positively regulates the synthesis of flavonoids in licorice cells.

With the increasing market demand and overexploitation, the availability of wild licorice has been greatly reduced and cannot meet the current demand. As an alternative, cell suspension systems are generally considered a suitable method for large-scale production [5], and this research shows that exogenous MeJA can be used to activate TF-related genes and increase gene expression related flavonoid biosynthesis. The transcriptome data is also an important public resource that could be used to accelerate the research of the MeJA response network and the regulatory mechanism involved in plant flavonoid biosynthesis. With the increasing knowledge of MYB TFs and their regulation of flavonoid biosynthesis, there exists a real possibility for mass production of flavonoids via direct genetic manipulation to increase the productive capacity of cell cultures.

## Supporting information

S1 Fig The establishment of the suspended licorice cell lines. (TIF)

S2 Fig The NR comparison results. (TIF)

S3 Fig Volcano plot. (TIF)

S4 Fig Gene clone (A) *GlMYB4*; (B) *GlMYB88*. (TIF)

S5 Fig pJG054 vector map. (JPG)

S6 Fig Kanamycin resistance concentration screening. (TIF)

S7 Fig Detecting of transformed cell vector marker gene. (TIF)

S8 Fig The rutin standard curve. (TIF)

S9 Fig The isoliquiritigenin standard curve. (TIF)

S1 Table MeJA treatment conditions. MeJA is the experimental group, and anhydrous ethanol represents the control group.
(DOCX)

S2 Table Primer sequence. (DOCX)

S3 Table Statistical table of the splicing of transcripts. (DOCX)
