# Supplementary figures and images for: Two responses to MeJA induction of R2R3-MYB transcription factors regulate flavonoid accumulation in Glycyrrhiza uralensis Fisch
Source: PLoS One. 2020 Jul 30;15(7):e0236565. doi: 10.1371/journal.pone.0236565 (PMC7392228; doi:10.1371/journal.pone.0236565)

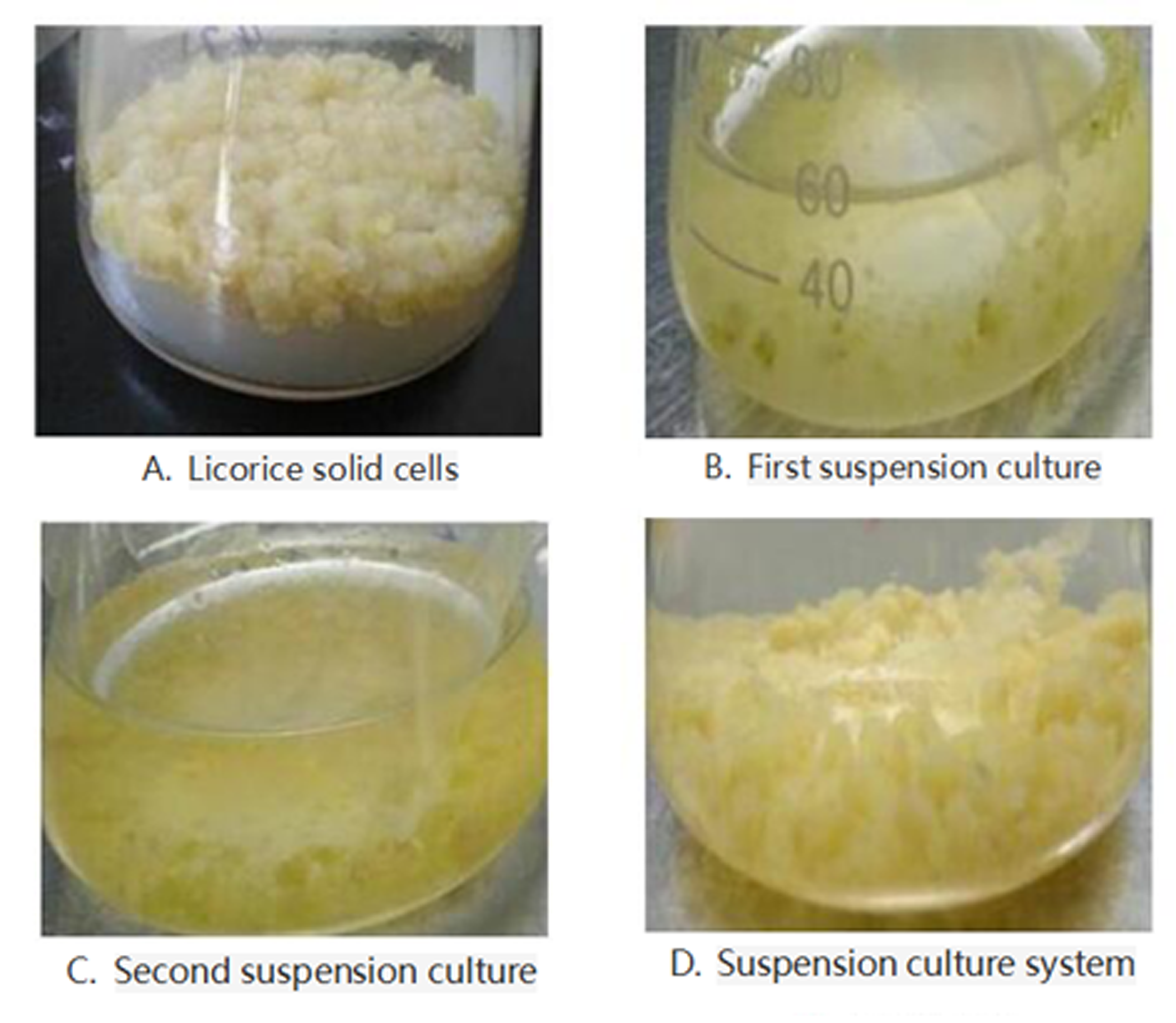

Supplement: S1 Fig — (TIF) [file pone.0236565.s001.tif]

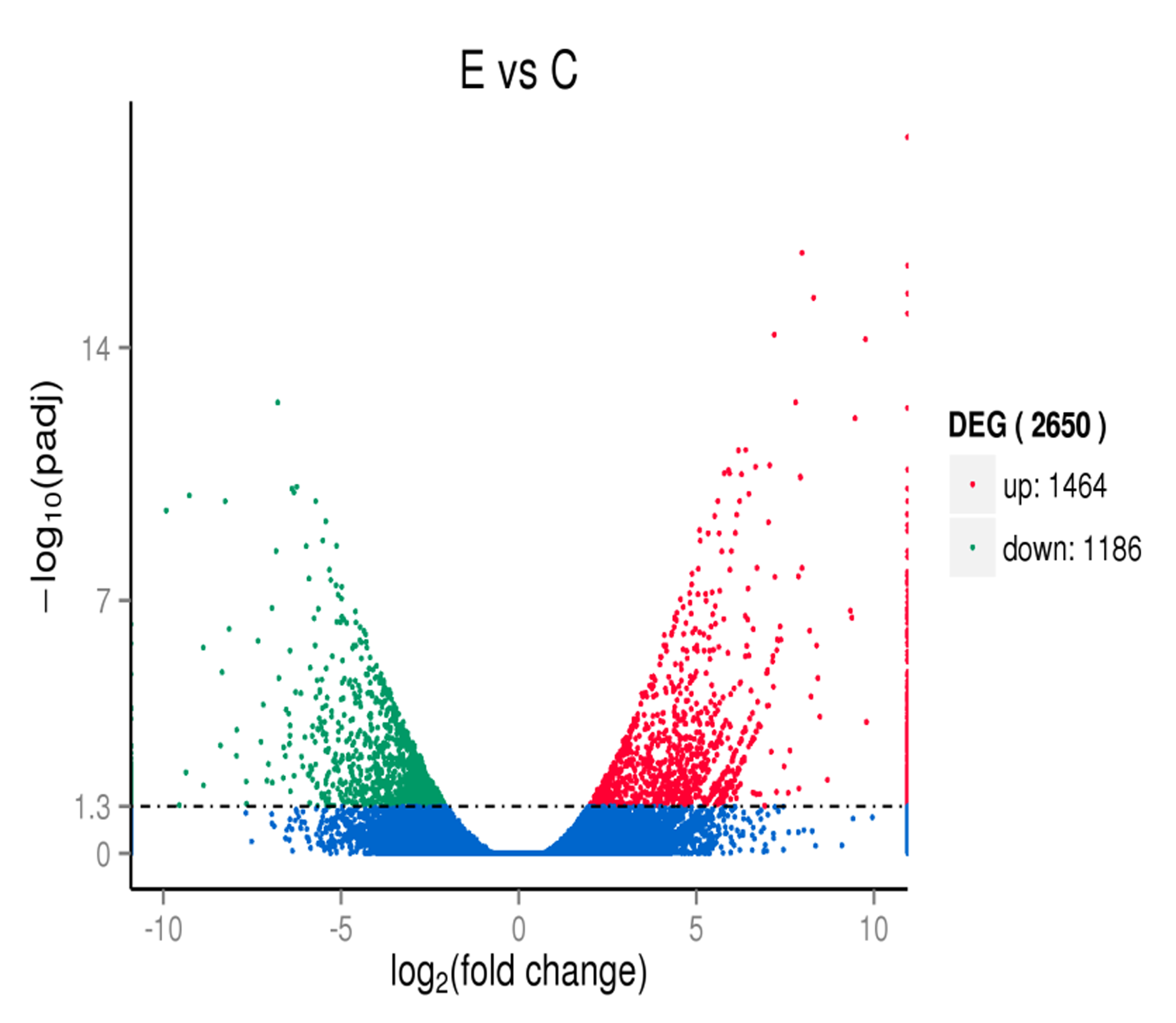

Supplement: S3 Fig — (TIF) [file pone.0236565.s003.tif]

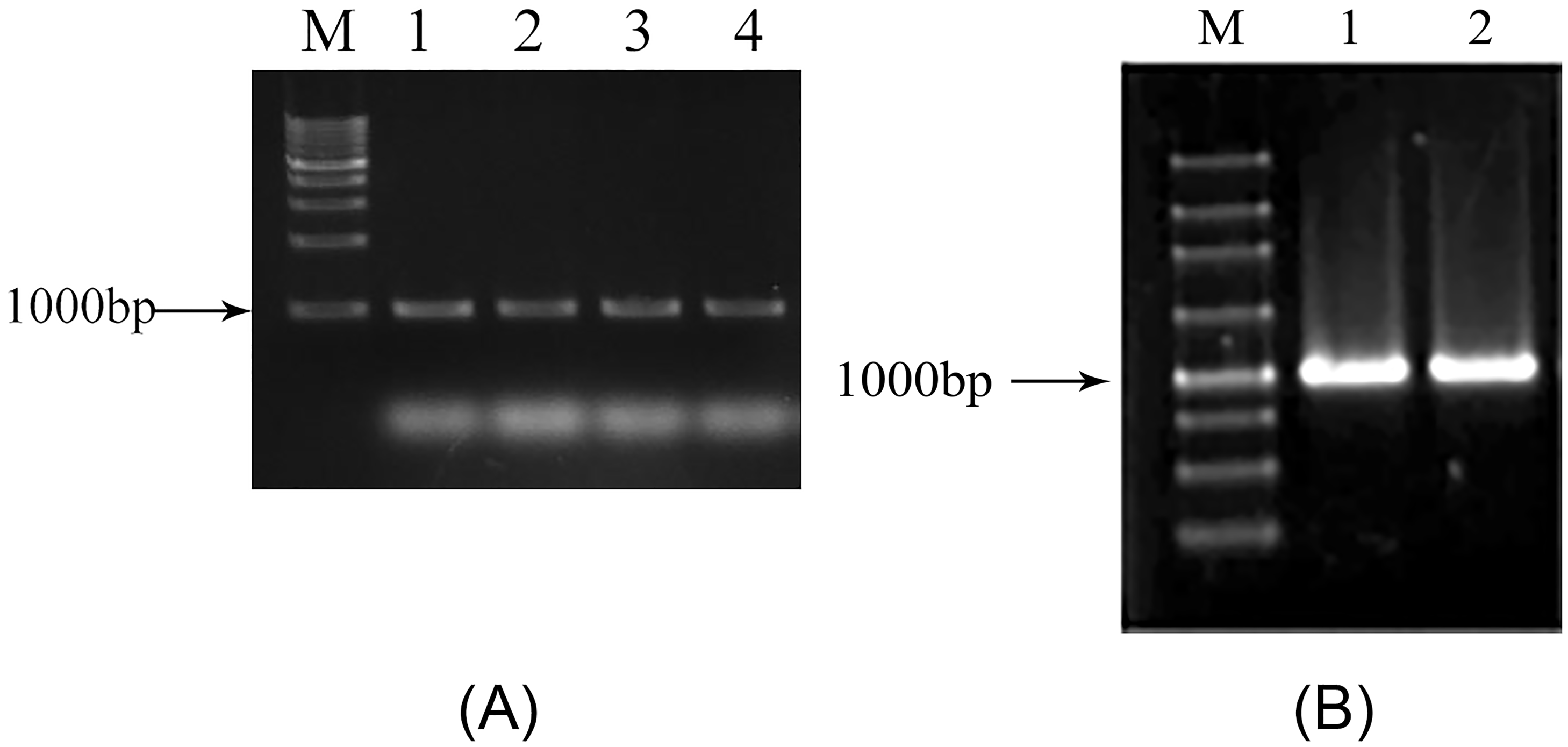

Supplement: S4 Fig — (TIF) [file pone.0236565.s004.tif]

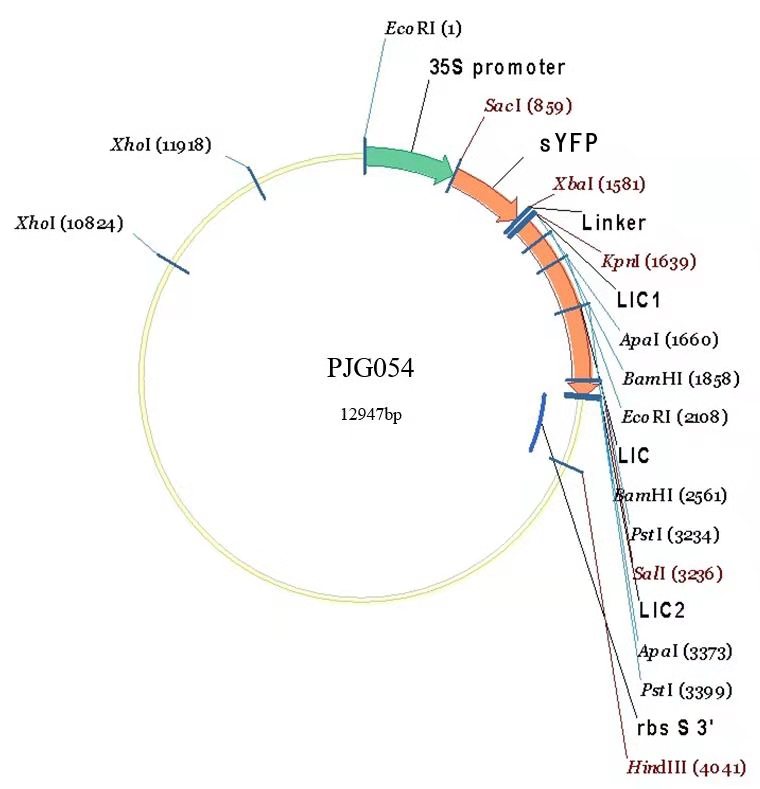

Supplement: S5 Fig — (JPG) [file pone.0236565.s005.jpg]

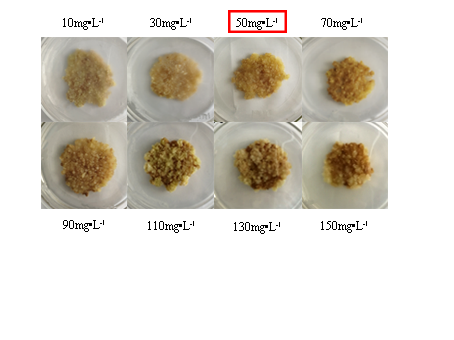

Supplement: S6 Fig — (TIF) [file pone.0236565.s006.tif]

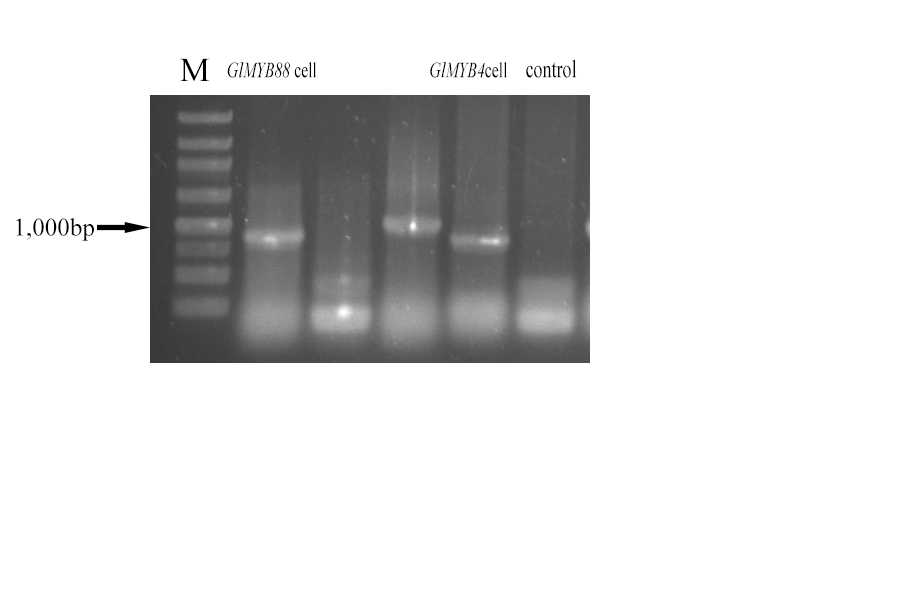

Supplement: S7 Fig — (TIF) [file pone.0236565.s007.tif]
