# Supplementary material for: Two responses to MeJA induction of R2R3-MYB transcription factors regulate flavonoid accumulation in Glycyrrhiza uralensis Fisch
Source: PLoS One. 2020 Jul 30;15(7):e0236565. doi: 10.1371/journal.pone.0236565 (PMC7392228; doi:10.1371/journal.pone.0236565)
Supplement: S1 Table — MeJA is the experimental group, and anhydrous ethanol represents the control group. (DOCX) [file pone.0236565.s010.docx]

| Sample | Adding MeJA solution (μL) | Adding anhydrous ethanol (μL) | Processing time (h) |
| --- | --- | --- | --- |
| 1 | 25 | 0 | 1 |
| 2 | 0 | 20 | 1 |
| 3 | 25 | 0 | 3 |
| 4 | 0 | 20 | 3 |
| 5 | 25 | 0 | 6 |
| 6 | 0 | 20 | 6 |
| 7 | 25 | 0 | 9 |
| 8 | 0 | 20 | 9 |
| 9 | 25 | 0 | 12 |
| 10 | 0 | 20 | 12 |
