# Supplementary material for: Two responses to MeJA induction of R2R3-MYB transcription factors regulate flavonoid accumulation in Glycyrrhiza uralensis Fisch
Source: PLoS One. 2020 Jul 30;15(7):e0236565. doi: 10.1371/journal.pone.0236565 (PMC7392228; doi:10.1371/journal.pone.0236565)
Supplement: S2 Table — (DOCX) [file pone.0236565.s011.docx]

| Gene | Primer sequence | |
| --- | --- | --- |
|  | Forward primer（5’-3’） | Reverse primer（5’-3’） |
| CHS | GAGAACAACAAAGGTGCTCGTG | GCCGTCCAAACCAACTCAAAT |
| C4H | ACATTTTGGACGCTCAGAAGAA | ATCTGGCTCAGTCACTTGGTGT |
| Actin | AGAAGGATGCCTATGTGGGTG | TCTGTTTGCTTTTGGGTTGAG |
| GlMYB4 | AGAGACCCATAATTGCACATGC | TTATGTTTCCATGCCCTTTTGA |
| J4 | AAGGGCCCTTATGATCGATGATATCCCATGGGC | AAAGGGCCCTTTACTCGAGGGATCCAGATCTCCAG |
| M4D1 | AATACGACTCACTATAGGGCGAC | CTTCTTCAAACCCATCTTCTCAC |
