# Supplementary material for: Two responses to MeJA induction of R2R3-MYB transcription factors regulate flavonoid accumulation in Glycyrrhiza uralensis Fisch
Source: PLoS One. 2020 Jul 30;15(7):e0236565. doi: 10.1371/journal.pone.0236565 (PMC7392228; doi:10.1371/journal.pone.0236565)
Supplement: S3 Table — (DOCX) [file pone.0236565.s012.docx]

|  | Reads | Transcripts | Unigenes |
| --- | --- | --- | --- |
| E-1 | 50291136 | 151529 | 116907 |
| E-2 | 42257958 |  |  |
| E-3 | 54019220 |  |  |
| C-1 | 43148884 |  |  |
| C-2 | 50834752 |  |  |
| C-3 | 49819038 |  |  |
